# Dosimetric comparison for volumetric modulated arc therapy and intensity-modulated radiotherapy on the left-sided chest wall and internal mammary nodes irradiation in treating post-mastectomy breast cancer

**DOI:** 10.2478/raon-2014-0033

**Published:** 2015-03-03

**Authors:** Qian Zhang, Xiao Li Yu, Wei Gang Hu, Jia Yi Chen, Jia Zhou Wang, Jin Song Ye, Xiao Mao Guo

**Affiliations:** 1 Department of Radiation Oncology, Fudan University Shanghai Cancer Center; Department of Oncology, Shanghai Medical College, Fudan University, 270 Dong An Road, Shanghai 200032, China; 2 Swedish Cancer Institute, 747 Broadway, Seattle, USA

**Keywords:** breast cancer, radiotherapy, VMAT, IMRT

## Abstract

**Background:**

The aim of the study was to evaluate the dosimetric benefit of applying volumetric modulated arc therapy (VMAT) on the post-mastectomy left-sided breast cancer patients, with the involvement of internal mammary nodes (IMN).

**Patients and methods:**

The prescription dose was 50 Gy delivered in 25 fractions, and the clinical target volume included the left chest wall (CW) and IMN. VMAT plans were created and compared with intensity-modulated radiotherapy (IMRT) plans on Pinnacle treatment planning system. Comparative endpoints were dose homogeneity within planning target volume (PTV), target dose coverage, doses to the critical structures including heart, lungs and the contralateral breast, number of monitor units and treatment delivery time.

**Results:**

VMAT and IMRT plans showed similar PTV dose homogeneity, but, VMAT provided a better dose coverage for IMN than IMRT (p = 0.017). The mean dose (Gy), V_30_ (%) and V_10_ (%) for the heart were 13.5 ± 5.0 Gy, 9.9% ± 5.9% and 50.2% ± 29.0% by VMAT, and 14.0 ± 5.4 Gy, 10.6% ± 5.8% and 55.7% ± 29.6% by IMRT, respectively. The left lung mean dose (Gy), V_20_ (%), V_10_ (%) and the right lung V_5_ (%) were significantly reduced from 14.1 ± 2.3 Gy, 24.2% ± 5.9%, 42.4% ± 11.9% and 41.2% ± 12.3% with IMRT to 12.8 ± 1.9 Gy, 21.0% ± 3.8%, 37.1% ± 8.4% and 32.1% ± 18.2% with VMAT, respectively. The mean dose to the contralateral breast was 1.7 ± 1.2 Gy with VMAT and 2.3 ± 1.6 Gy with IMRT. Finally, VMAT reduced the number of monitor units by 24% and the treatment time by 53%, as compared to IMRT.

**Conclusions:**

Compared to 5-be am step-and-shot IMRT, VMAT achieves similar or superior target coverage and a better normal tissue sparing, with fewer monitor units and shorter delivery time.

## Introduction

Among the most commonly diagnosed cancers, breast cancer alone accounts for 29% of all new cancers among women in 2014.^1^ Most early-stage patients can be treated with breast conserving surgery, adjuvant radiotherapy or systemic treatment combined with neoadjuvant chemotherapy.^2^ However, patients with the advanced conditions usually receive mastectomy and postoperative radiotherapy. It has been shown that adjuvant post mastectomy radiotherapy (PMRT) is efficient in reducing locoregional recurrence rate, and improving 10-year overall survival rate in patients with lymph node-positive breast cancer.^3^,^4^–^8^

However, there is a dosimetric challenge to deliver an uniform target dose to the patient with three-dimensional conformal radiotherapy (3D-CRT) if internal mammary node (IMN) is involved, especially in the patients with left-sided breast cancer.^9^,^10^ In order to achieve better cosmetic results and decrease the toxicity in normal tissues, the intensity modulated radiation therapy (IMRT) has been widely implemented in the clinic to improve the target dose homogeneity and conformity for breast cancer treatment as well as spare the irradiation doses of normal tissues.^11^–^13^ Compared to the 3D-CRT, Van der Laan *et al*. reported that the IMRT technique improved the chest wall (CW) and IMN dose coverage and reduced the cardiac dose. Previously, we conducted a similar study in 30 patients with left-sided post-mastectomy breast cancer, and the results showed that the conformity index of IMRT was better than that of 3D-CRT and IMRT increased the low-dose volume of normal tissue.^14^,^15^

Volumetric modulated arc therapy (VMAT), a novel technique that delivers the radiation dose to the target in a single or multiple gantry rotations, has been used in the treatment of many cancers sites, such as prostate, head and neck, and Hodgkin lymphoma.^16^–^20^ Some dosimetric studies compared VMAT with other techniques in treating breast cancer patients.^21^,^22^ Also, one study compared the rapid arc (a VMAT technique), IMRT and modified wide-tangent techniques in the left-sided breast cancer and found that the rapid arc could achieve similar target coverage as IMRT but with better organ at risks sparing and shorter treatment time, though only one patient received mastectomy in their 5-patient study.^23^ To master the application of VMAT with better efficacy, we investigated the dosimetric difference between the VMAT and IMRT in patients with left-sided breast cancer in the present study.

## Patients and methods

### Patients

From April 2009, the first fifteen left-sided breast cancer patients (T3/4, metastatic axillary lymph nodes > 4) treated in our department, with the mean age of 48 years (39 to 58), were enrolled in the study. All patients had undergone post-mastectomy and Level I–II nodal dissection and received the combined chemotherapy with or without trastuzumab. Patients were set up on a breast board (Med-Tec Corporation, USA) with the sternum parallel to the table and the left arms elevated above their heads. The patient’s head turned to the right side. The radio-opaque markers were placed on the patient’s midline, mid axillary line, the inferior aspect of the clavicle head, the inferior border at 1 cm below the contralateral infra mammary fold and the superior aspect of the fourth rib. CT images were acquired from the level of mandible to the lung base on a large bore CT scanner (Philips Medical, Fitchburg, WI, USA) with a slice thickness of 5 mm. All the images were exported to the Pinnacle treatment planning system (Pinnacle^3^ version 9.0, Philips Radiation Oncology Systems, Andover, MA) for contouring and treatment planning.

### Target definitions

The clinical target volume (CTV) of CW (CTV_CW_) and IMN (CTV_IMN_) was delineated according to the Radiation Therapy Oncology Group (RTOG) breast cancer consensus definitions. The CTV_IMN_ was contoured from the superior aspect of the medial first rib to the forth one by encompassing the internal mammary/thoracic vessels. A margin of 10 mm was added to CTV_CW_ and CTV_IMN_ to define the planning target volume of CW (PTV_CW_) and IMN (PTV_IMN_). Total PTV (PTV_total_) consisted of PTV_CW_ and PTV_IMN_. All the PTV_CW_, PTV_IMN_ and PTV_total_ were limited to the skin surface. The organs at risk were also outlined: the heart contoured from the first CT slice below the pulmonary artery to the apex inferiorly; the entire ipsilateral and contralateral lung contoured; and the contralateral breast outlined based on the visible breast parenchyma.

### Treatments

The treatments were planned for delivery on an Elekta Synergy linear accelerator (Elekta Oncology System, Crawley, UK) with 1-cm width multileaf collimator (MLC). A 5 mm tissue-equivalent bolus was placed on the patient’s skin with the coverage of PTV and surgical scar to increase the skin dose. The dose was calculated using the collapse cone superposition convolution algorithm with inhomogeneity correction.

In the present dosimetric study, one step-and-shoot IMRT and one VMAT treatment plan were created for each patient within the Pinnacle treatment planning system with the same dose optimization objectives. The isocenter was placed at the center of the PTV. The prescription dose was 50 Gy in 25 fractions. The plan quality for both treatment techniques was evaluated against the following criteria: at least 95% of the PTV volume receiving 50 Gy, 95% of the prescription dose (V_95%_) covering at least 99% of the PTV volume; the hot spot defined as PTV receiving more than 110% of prescription dose as little as possible; less than 20% of the left lung to 20 Gy (V_20_); less than 10% of the heart to 30 Gy (V_30_); a minimized dose to the contralateral lung and breast since some of the beams could penetrate the patient’s right lung and right breast.

A step-and-shoot IMRT plan with 5 beams (300, 0, 40, 80 and 110 degree) was created for each patient. The optimization was performed using the direct machine parameter optimization (DMPO) technique with preset parameters of minimum 3 monitor units, minimum 3 cm^2^ segment area and maximum 50 segments. Before the final dose calculation, the MLC leaves were manually pushed outside of the patient’s skin by 1 cm if they blocked only the air part in the beam’s eye view.

The SmartArc in Pinnacle was used for the VMAT planning. One or two 200 degree partial arcs (gantry rotated from 310 to 150 degrees) and 15 degree collimator rotation were utilized to generate VMAT plans. A 4-degree resolution was used for the final dose calculation. For the purpose of fair plan comparison, several step-and-shoot IMRT and SmartArc VMAT plans were created for the initial 3 patients and the best IMRT and VMAT plans were selected for dose volume histogram (DVH) data analysis. Then, the optimization parameters for the best plans were used for the following patients and all the required DVH data were obtained.

The DVHs of the PTV_total_, PTV_IMN_, lungs, heart and contralateral breast were derived from the IMRT and VMAT plans. For the targets were calculated the D_98_ (the minimum dose received by 98% of the target volume), D_2_, mean dose, dose homogeneity index (HI), V_90%_ (percentage of the PTV receiving at least 90% of the prescription dos e) and V_95%_. D_98_ and D_2_ were used to evaluate the minimal and maximal dose to the target, respectively. The homogeneity index was calculated as follows:

$$\mathrm{HI}=\frac{\left(D_{2}-D_{98}\right)}{D_{P}}*100\%$$

where the D_p_ is the prescription dose, and lower HI means better homogeneity. Additionally, the V_110%_ and V_115%_ for the PTV_IMN_ were also recorded. For the critical structures, the mean dose, V_30_, V_5_, and V_10_ of the heart, and V_20_, V_5_, V_10_ and mean dose of the ipsilateral lung, V_5_ and mean dose of the contralateral lung and mean dose of the contralateral breast were calculated. Number of monitor units and treatment delivery time were also calculated. Dry runs were performed for all the plans.

### Statistical analysis

The results were represented as mean ± standard deviation (SD). Statistical analysis was performed using SPSS 17.0 software (Chicago, IL, USA). The two-sided paired t test was used when the data-sets were normally distributed. Otherwise, datasets were compared by Wilcoxon Cox test. The p value less than 0.05 was considered statistically significant.

## Results

### Target coverage

**FIGURE 1.**
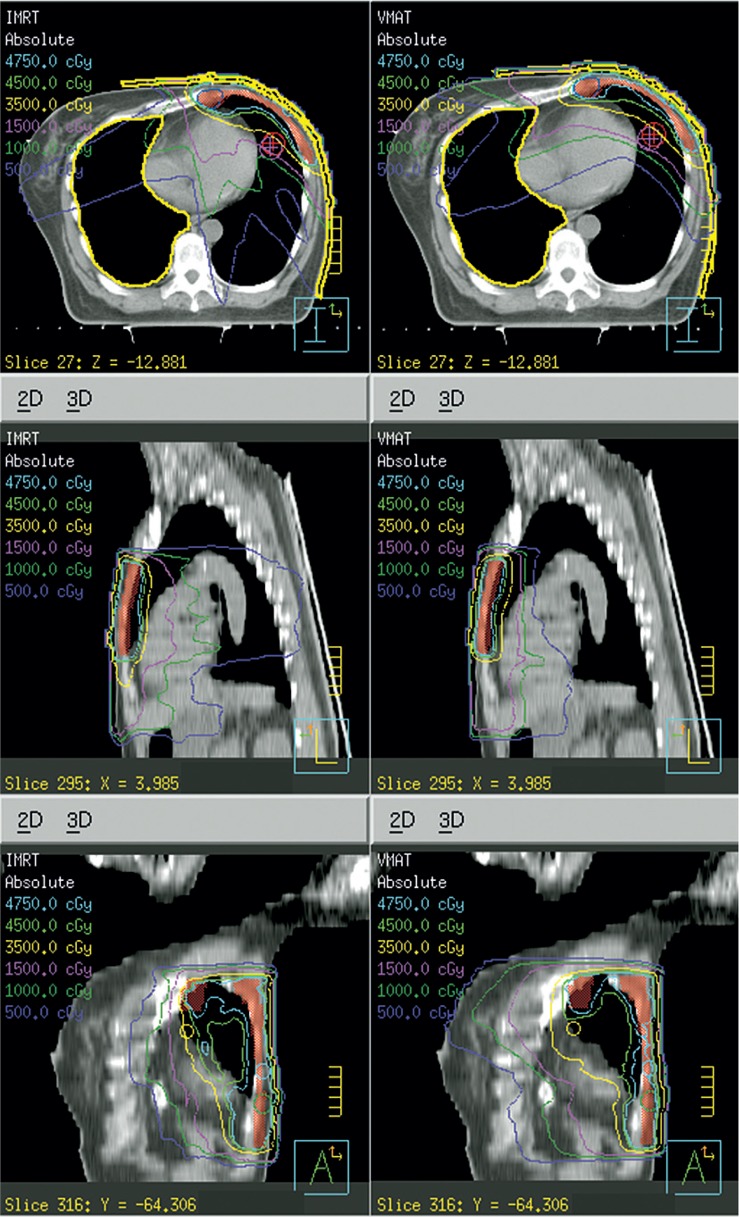
Comparison between volumetric modulated arc therapy (VMAT) and intensity-modulated radiotherapy (IMRT) on dose distribution on the transverse plane at isocenter (from one representative case). The VMAT plan is on the right side and the IMRT on the left side.

**FIGURE 2.**
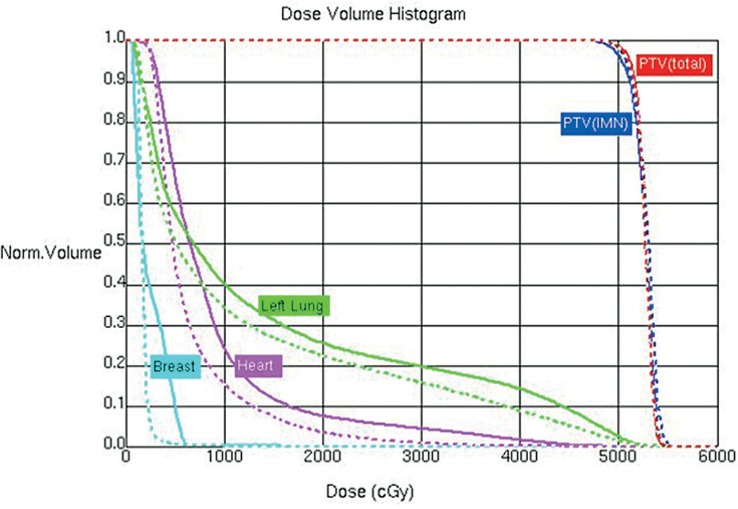
Comparison between volumetric modulated arc therapy (VMAT) and intensity-modulated radiotherapy (IMRT) on dose volume histogram for PTV_total_, PTV_IMN_, heart, left lung and the contralateral breast (from one representative case shown in Figure 1). The VMAT plan is displayed as dashed line, IMRT plan as solid line.

The mean volume of PTV_total_ was 212cm^3^ (90 to 425 cm^3^) . A dose distribution is shown in Figure 1 and the corresponding DVHs in Figure 2 for a typical patient. The differences in the PTV_total_ coverage and dose homogeneity between two techniques were of no statistical significance, V_95%_ being 99.1% ± 1.1% with VMAT and 98.9% ± 1.1% with IMRT (p = 0.363); the similar maximum dose of PTV_total_ defined as one in 2% of the target volume, i.e. D_2_, 55.6 ± 2.2 Gy with IMRT and 55.4 ± 1.7 Gy with VMAT, respectively; and the dose homogeneity index being 0.15 with both VMAT and IMRT (Table 1).

As for the dosimetric comparison data for the smaller PTV_IMN_, the VMAT plans provided a better IMN coverage than the IMRT ones, the mean values of V_95%_ were 99.2% ± 1.8% and 98.1% ± 2.9% with VMAT and IMRT, respectively (p = 0.017). Although there was no significant difference in PTV_IMN_ mean doses, the VMAT plans seemed to develop more homogeneous dose distribution in the IMN. The minimal dose to PTV_IMN_ (D_98_) with VMAT was higher than that with IMRT (45.3 ± 6.9 Gy for VMAT vs 41.7 ± 5.4 Gy for IMRT) (p = 0.016). The mean HI was found to be 0.13 ± 0.06 with VMAT and 0.15 ± 0.06 with IMRT (p = 0.048). Both techniques presented comparable hot spots as the p values for V_110%_ and V_115%_ were 0.421 and 0.334, respectively (Table 1).

### Normal tissue sparing

In terms of the doses to the normal tissues for the two treatment techniques, VMAT slightly reduced the mean dose to the heart, 13.5 ± 5.5 Gy for VMAT vs. 14.0 ± 5.3 Gy for IMRT (p = 0.792). Meanwhile, it did not show any significant differences in heart V_30_ and V_5_, as well as in V_10_ compared with IMRT (50.2% ± 29.0% with VMAT vs. 55.7% ± 29.6% with IMRT, p = 0.611) (Table 2).

It was also found that the VMAT plans achieved lower mean dose to the left lung than the IMRT ones, *i.e*., 12.8 ± 1.9 Gy vs. 14.1 ± 2.3 Gy (p = 0.001). Moreover, the values of left lung V_20_, V_10_ and V_5_ were 21.0% ± 3.8%, 37.1% ± 8.4%, 61.1% ± 18.0% for VMAT, and 24.2% ± 5.9%, 42.4% ± 11.9 %, 66.0% ± 15.5% for IMRT. There was no significant difference in the mean dose of right lung, but VMAT plans achieved lower V_5_ to the right lung, as compared to IMRT (32.1% ± 18.2% with VMAT vs. 41.2% ± 12.3% with IMRT, p = 0.034). The mean dose to the contralateral breast was 1.7 ± 1.2 Gy and 2.3 ± 1.6 Gy, respectively (p = 0.001) (Table 2).

### Monitor units and treatment delivery time

The dose rate for IMRT was 512 MU/min, and the maximum dose rate for VMAT was 512 MU/min. The mean number of MU for VMAT plans was 462 (range, 380 to 590 MU) compared to 604 (range, 488 to 850 MU) for IMRT. The mean treatment time for one arc was 2.0 minutes, and the mean treatment time to deliver two arcs was 4.20 minutes (range, 4.1 to 4.3 minutes) compared to 9.0 minutes (range, 8.7 to 11.2 minutes) for IMRT.

## Discussion

IMRT and VMAT can shape the dose to the concave target in the CW and IMN in breast cancer radiotherapy. In the current study, we reported a dosimetric comparison between the two techniques on 15 cases of left-sided breast cancer. The step-and-shoot IMRT plans using DMPO technique and the VMAT plans using the SmartArc were used in the Pinnacle treatment planning system. In our study, CT images were acquired base on a CT scanner with a slice thickness of 5 mm. Though the widths of slices are usually 2–3 mm, CT scan could also be performed using 5 mm slice thickness to evaluate the dose distribution of IMRT3.^3^–^7^

### Target coverage

It has a benefit in maximizing efficacy and improving local control to ensuring homogeneous dose coverage of PTV by avoiding areas of under dose (‘cold spots’, PTV receiving less than 90% of prescription dose), and at the same time eliminating areas of relative overdose (‘hot spots’), minimizing normal long-term tissue toxicity (skin changes and fibrosis) which negatively affect cosmesis. In our study, the IMRT and VMAT plans showed similar PTV_total_ coverages and both avoided the hot spots successfully. However, the VMAT had a better dose homogeneity in the PTV_IMN_ by reducing the “cold spot”, which might decrease the local recurrence in the IMN area.

The radiotherapy target volume includes the CW, sup raclavicular fossa and IMN with or without the axilla.^24^,^25^ Though the inclusion of the supraclavicular region in the post-mastectomy radiotherapy has an influence on the dose to the ipsilateral lung, it is still reasonable and significant to compare the dose coverage between IMRT and VMAT when the supraclavicular region was not considered for all the patients. On the other hand, PTV should be a few millimetres below the skin surface. In our study, the PTVs were limited to the skin surface due to that chest has thinner wall with a few millimetres and bolus was placed on the patient’s chest skin surface to increase the skin dose. Therefore, the skin could provide the dose we needed and it is unnecessary to subtract a few millimetres from the skin surface.

### Organs at risk dose

It has been shown that the V_20_ and mean dose to the lung are good predictors for radiation induced lung toxicity.^26^ Also, an analysis of non-small-cell lung cancer has shown that the V_5_ is a significant cut off point for the subsequent development of pneumonitis.^27^ When it comes to the breast cancer, it was found that clinically significant pneumonitis was rare if the V_20_ of ipsilateral lung was less than 30% for breast cancer patients.^28^ It has been also reported that the complication rate could be expected to be 20% if more than 50% of the lung volume received 10 Gy.^29^ We selected V20 < 20% as a criterion since it is also an optimization parameter in our centre. We found that the VMAT plan had a significant reduction in the V_20_, V_10_, V_5_ or the mean dose in the left lung than IMRT. Also, the VMAT showed the superior or similar right lung sparing compared with IMRT. These results strongly suggested that the VMAT technique could achieve better sparing of the lung.

It has been reported that the use of 3D-CRT and IMRT techniques in the treatment of breast cancer could reduce the cardiac dose and cardiac mortality.^30^,^31^ However, the potential cardiac toxicity was increased dramatically owing to the widespread use of anthracyclines, taxanes and trastuzumab.^32^,^33^ Therefore, it is critical to limit the heart dose in patients, especially those with left-sided breast cancer. It has been reported that the heart V30 of IMRT was significantly lower than 3D-CRT levels for patients underwent left- sided mastectomy.^14^ Rudat *et al.* have found that IMRT significantly reduced the ipsilateral lung dose and heart dose in 20 subsequent post mastectomy breast cancer patients.^34^ Moreover, VMAT has been revealed to deliver lower doses to the ipsilateral breast and lung and offer better dose conformity than 3D-CRT technique for partial breast irradiation patients.^35^ In this study, the dose to the heart for IMRT and VMAT was similar.

The dose to the contralateral breast is another critical factor to consider, especially in younger women who received RT. Previous studies showed that there was an elevated long-term risk of developing the secondary contralateral breast cancer, and the mean dose to the contralateral breast was 3.2 Gy with RapidArc.^23^,^36^ In our study, a slightly lower mean dose of 1.7 Gy was observed with VMAT, which may be the results of different dose calculation algorithms or inhomogeneity correction in the two treatment planning systems. We also found that the average mean dose to the contralateral breast was 2.3 Gy in the IMRT, suggesting that VMAT might have dosimetric effect in reducing the risk of contralateral breast cancer occurrence.

### Organ motion

It is well known that the respiration-induced target motion can lead to variation between the planned and delivered dose. A 10 mm margin was applied in the study for expanding the CTV to PTV. We then evaluated the intra-fraction motion of the chest wall using the fluoroscopic imaging on the simulator and found that the maximum displacement was around 3 mm. It’s been reported that the respiratory movements of the breast during normal breathing were negligible, and at 80% of the tidal capacity the mean displacement of the breast and chest wall from the exhale was less than 1 mm in the anterior and superior directions.^37^,^38^ The 5 mm margin may extend the PTV to the outside of skin. With limited segments of the step-and-shot IMRT plans (maximum 50 segments), the MLC leaves can be pushed outwards from the patient’s skin by 1 or 2 cm if only the air part in BEV was blocked. However, such manual adjustment is unfeasible in the VMAT plans. Therefore, the solutions with improved target coverage for possible changes in size and position of target and rest tissues caused by respiration or oedema are to use the third-party software to move the block-air MLC away from the skin, or manually add 10-mm tissue around the skin for optimization but removing it in the final dose calculation.^39^ Another clinical advantage of VMAT is that it generally takes fewer MUs to deliver a VMAT treatment than IMRT for the same plan quality. Our results showed that the MUs for the fifteen chest walls examined by VMAT plans were about 2/3 to 3/4 of those by IMRT plans. Obviously, fewer MUs are always favourable as to shorten the treatment delivery time and reduce the whole body dose.

## Conclusions

Overall, our results showed that VMAT achieved similar or superior target coverage, better normal tissue sparing, fewer monitor units and shortened delivery time, as compared with 5-beam step-and-shot IMRT.

## Figures and Tables

**TABLE 1.** Comparison of the dose coverage for the PTV_total_ and the PTV_IMN_ (mean ± SD)

**PTV_total_**		**IMRT**	**VMAT**	**p value**
Max dose (D_2_)	(Gy)	55.6 ± 2.2	55.4 ± 1.7	0.760
Min dose (D_98_)	(Gy)	48.8 ± 1.0	48.5 ± 2.2	0.616
Mean dose	(Gy)	52.6 ± 1.2	52.4 ± 1.7	0.344
HI		0.15 ± 0.05	0.15 ± 0.01	0.602
V_45_	(%)	99.8 ± 0.3	100.0 ± 0.1	0.524
V_47.5_	(%)	98.9 ± 1.1	99.1 ± 1.1	0.363
PTV_IMN_				
Max dose (D_2_)	(Gy)	56.8 ± 2.0	56.2 ± 1.6	0.126
Min dose (D_98_)	(Gy)	41.7 ± 5.4	45.3 ± 6.9	0.016
Mean dose	(Gy)	52.6 ± 1.8	53.1 ± 1.1	0.207
HI		0.15 ± 0.06	0.13 ± 0.06	0.048
V_45_	(%)	99.3 ± 1.5	100.0 ± 0.1	0.017
V_47.5_	(%)	98.1 ± 2.9	99.2 ± 1.8	0.017
V_55_	(%)	14.6 ± 24.6	15.7 ± 19.9	0.787
V_57.5_	(%)	4.0 ± 16.3	2.0 ± 3.6	0.421

HI = homogeneity index; IMRT = intensity-modulated radiotherapy; Max = maximal; Min = minimal; PTV_IMN_ = internal mammary node planning target volume; PTV_total_ = planning target volume; SD = standard deviation; V_45_ = the percentage of the lung volume which receives radiation doses of 45 Gy; VMAT = volumetric modulated arc therapy

**TABLE 2.** Comparison parameters of normal tissue with VMAT or IMRT (mean ± SD)

**Structure**	**Parameters**		**IMRT**	**VMAT**	**VMAT/IMRT**	**p value**
Heart	Mean dose	(Gy)	14.0 ± 5.3	13.5 ± 5.0	0.97 ± 0.05	0.792
	V_30_	(%)	10.6 ± 5.8	9.9 ± 5.9	0.91 ± 0.30	0.251
	V_10_	(%)	55.7 ± 29.6	50.2 ± 29.0	0.89 ± 0.12	0.611
	V_5_	(%)	77.0 ± 21.1	78.0 ± 20.1	1.02 ± 0.06	0.355
Left Lung	Mean dose	(Gy)	14.1 ± 2.3	12.8 ± 1.9	0.91 ± 0.05	0.001
	V_20_	(%)	24.2 ± 5.9	21.0 ± 3.8	0.89 ± 0.09	0.002
	V_10_	(%)	42.4 ± 11.9	37.1 ± 8.4	0.89 ± 0.09	0.001
	V_5_	(%)	66.0 ± 15.5	61.1 ± 18.0	0.92 ± 0.07	0.001
Right Lung	Mean dose	(Gy)	4.67 ± 0.93	4.49 ± 1.06	0.94 ± 0.14	0.409
	V_5_	(%)	41.2 ± 12.3	32.1 ± 18.2	0.71 ± 0.31	0.034
Right Breast	Mean dose	(Gy)	2.3 ± 1.6	1.7 ± 1.2	0.70 ± 0.04	0.002

IMRT = intensity-modulated radiotherapy; SD = standard deviation; V_20_ = the percentage of the lung volume which receives radiation doses of 30 Gy; VMAT = volumetric modulated arc therapy
